# Impact of Superstorm Sandy on Medicare Patients’ Utilization of Hospitals and Emergency Departments

**DOI:** 10.5811/westjem.2017.7.34730

**Published:** 2017-09-21

**Authors:** Benoit Stryckman, Lauren Walsh, Brendan G. Carr, Nathaniel Hupert, Nicole Lurie

**Affiliations:** * U.S. Department of Health and Human Services, Washington, D.C.; † Thomas Jefferson University, Department of Emergency Medicine, Philadelphia, Pennsylvania; ‡ Weill Cornell Medical College, Department of Healthcare Policy and Research, Department of Medicine, New York, New York

## Abstract

**Introduction:**

National health security requires that healthcare facilities be prepared to provide rapid, effective emergency and trauma care to all patients affected by a catastrophic event. We sought to quantify changes in healthcare utilization patterns for an at-risk Medicare population before, during, and after Superstorm Sandy’s 2012 landfall in New Jersey (NJ).

**Methods:**

This study is a retrospective cohort study of Medicare beneficiaries impacted by Superstorm Sandy. We compared hospital emergency department (ED) and healthcare facility inpatient utilization in the weeks before and after Superstorm Sandy landfall using a 20% random sample of Medicare fee-for-service beneficiaries continuously enrolled in 2011 and 2012 (N=224,116). Outcome measures were pre-storm discharges (or transfers), average length of stay, service intensity weight, and post-storm ED visits resulting in either discharge or hospital admission.

**Results:**

In the pre-storm week, hospital transfers from skilled nursing facilities (SNF) increased by 39% and inpatient discharges had a 0.3 day decreased mean length of stay compared to the prior year. In the post-storm week, ED visits increased by 14% statewide; of these additional “surge” patients, 20% were admitted to the hospital. The increase in ED demand was more than double the statewide average in the most highly impacted coastal regions (35% versus 14%).

**Conclusion:**

Superstorm Sandy impacted both pre- and post-storm patient movement in New Jersey; post-landfall ED surge was associated with overall storm impact, which was greatest in coastal counties. A significant increase in the number and severity of pre-storm transfer patients, in particular from SNF, as well as in post-storm ED visits and inpatient admissions, draws attention to the importance of collaborative regional approaches to healthcare in large-scale events.

## INTRODUCTION

National health security requires that healthcare facilities respond effectively and efficiently to disasters and public health emergencies. A key responsibility of the healthcare system is to anticipate, prepare for, and accommodate the increased demand for services following a catastrophic event, which is often referred to as “surge capacity.”^1^ Surge capacity is directly related to patient health outcomes. Patients admitted to the hospital during high surge periods have a significantly higher rate of mortality than those admitted in periods of low surge.

Further, emergency department (ED) crowding has been associated with increases in hospital length of stay for admitted patients.^2^ Increased hospital utilization after a disaster may result from illness or injury that is a direct effect of the event, as was the case following the 2013 Boston Marathon bombing or the 2016 Orlando nightclub shooting, or it may result from the movement of patients evacuated from a healthcare facility that can no longer care for them, as is common during severe flooding or sustained loss of electrical power. 3,4 While research has described methods for measuring surge capacity,^5^,^6^,^7^,^8^,^9^,^10^ little data exists that quantifies increased ED and inpatient utilization after major disasters. Consequently, the ability of receiving facilities to conduct evidence-based surge capacity planning is compromised.

Superstorm Sandy made landfall in New Jersey (NJ) on October 29, 2012, resulting in at least 37 deaths, 346,000 damaged or destroyed homes, and an estimated economic loss of $30 billion statewide.^11^,^12^,^13^ The NJ healthcare system was also directly affected, with two hospitals and many more nursing homes evacuated.^14^,^15^,^16^ This study analyzes hospital ED utilization patterns among fee-for-service (FFS) Medicare beneficiaries in NJ in the week before and after Superstorm Sandy’s landfall in order to (1) determine whether hospitals prepared for significant increases in healthcare demand by discharging patients early in the days preceding landfall, and (2) better characterize the impact of this storm as it relates to Medicare-patient surge and ED utilization.

## METHODS

### Study Population

We analyzed de-identified Medicare claims available from the Research Data Assistance Center (ResDAC)^17^ for ED utilization and patient disposition in New Jersey during Superstorm Sandy. We used this publicly available national data set to create a 20% random sample of FFS beneficiaries in NJ who were continuously enrolled from 2011 through 2012 (N=224,116). The percent of Medicare beneficiaries receiving FFS care in 2012 was 83.5%.^18^ We matched the study population to a comparison group made up of beneficiaries from non-Sandy-affected states (all U.S. states except FL, SC, NC, VA, DC, MD, DE, PA, NY, CT, RI, MA, NH, and VT).

We used the Community Hardship Index (CHI) to represent county-level storm impact. The index, developed by Rutgers University in the aftermath of Sandy,^19^ is comprised of weighted assessments of power loss; residential, commercial, and municipal damage; establishment of emergency shelters; and gasoline shortages, and controls for demographic differences in the population. Scores range from 1–100, with more severely impacted counties scoring higher.

Since this study involved the analysis of fully de-identified claims that cannot be traced back to the individual, it did not meet the definition of human subjects research as set forth in U.S. Code of Federal Regulations, and since there was no interaction or intervention with individuals and no identifiable private information used, it did not require review by an institutional review board.

**Population Research Capsule**

What do we already know about this issue?

While research has described methods for measuring surge capacity, there is a paucity of data quantifying increased ED utilization after major disasters.

What was the research question?

What is the magnitude and destination of the medical surge associated with a major natural disaster?

What was the major finding of the study?

In the week after Superstorm Sandy made landfall, ED visits by Medicare patients increased by 14% for the state of New Jersey.

How does this improve population health?

The evidence promotes population health resilience, improves healthcare delivery during disasters, and identifies patients at risk for adverse health outcomes.

### Key outcomes

Study outcomes were pre-storm hospital discharges, case-mix index (CMI), average length of stay (ALOS) for admitted patients, hospital admissions through the ED from inter-facility transfers; and post-storm ED visits, including those that resulted in a hospitalization (admissions) and those that did not (discharges). CMI is an average of the service intensity weights associated with diagnosis-related group (DRG) for a given population. CMI range is from 0.1 to 30.0. A higher CMI indicates more resources are needed to treat more complex patients.^20^ We identified inter-facility transfers using the hospital admission source variable or a hospital admission date falling between the admission and discharge dates of the transferring facility, and included patients who moved from hospital-to-hospital, hospice-to-hospital, and skilled nursing facility (SNF)-to-hospital.

### Data Analysis

#### Pre-Storm

We used chi-square statistics and logistic regression to examine the effect of Superstorm Sandy on pre-storm hospital discharges by comparing the number of discharges during the week prior to landfall (October 22, 2012 – October 28, 2012) to those during the equivalent week the previous year (October 24, 2011 – October 30, 2011), after adjusting for patient age, gender, and race. The same methods were used to calculate changes in pre-storm admissions through the ED from inter-facility transfers (hospital-to-hospital, hospice-to-hospital, and SNF-to-hospital).

We used t-test statistics and logistic regression to examine the effect of Superstorm Sandy on pre-storm CMI by comparing CMI of pre-storm hospital discharges and admissions from transfers the week prior to landfall to CMI of admissions from transfers on the equivalent week of the previous year. Ordinary least squares (OLS) was used to compute change in pre-storm ALOS, by comparing ALOS at discharge for matched DRGs the week prior to landfall to ALOS versus any other admission in 2011 and 2012 in NJ.

#### Post-Storm

We conducted two comparisons to assess post-storm ED utilization outcome measures among NJ Medicare FFS beneficiaries. First, we used a difference-in-differences (DD) regression to examine the effect of Superstorm Sandy on ED utilization among NJ beneficiaries by comparing the number of visits one week before landfall (October 22, 2012 – October 28, 2012) to utilization one week immediately after landfall (October 29, 2012 – November 4, 2012). This difference was then compared to the difference in visits between the equivalent weeks in the prior year (October 24, 2011 – October 30, 2011 and October 31, 2011 – November 6, 2011). The DD model was adjusted for patient age, gender, and race. Second, we used a difference-in-difference-in-differences (DDD) regression to test whether the storm’s effects on ED utilization were due to unobserved bias (e.g. seasonal influenza) among NJ beneficiaries rather than from the storm itself. 21 This was accomplished by implementing a 1,000-iteration Monte Carlo simulation (MCS) that randomly matched 1:1 (with replacement) demographic characteristics and post-storm ED utilization outcomes of beneficiaries’ cohorts in the NJ 2011 group from the first comparison to 2011 beneficiaries from states *not* impacted by Sandy. The Appendix includes a detailed description of the MCS process.

We conducted county-level analyses of storm impact by creating county-level quartiles based on the CHI associated with each county, with the first quartile (Q1) being the least severely impacted and the last quartile (Q4) being the most severely impacted counties (Table 1).

We performed all analyses using SAS software version 9.3 (SAS Institute Inc., Cary, NC). A two-sided P value of 0.05 or less was considered statistically significant.

## RESULTS

The NJ study population consisted of 224,116 continuously enrolled Medicare FFS beneficiaries. The study population represented approximately 2.5% of the 8.9 million NJ residents in 2012 and 16.0% of all NJ Medicare beneficiaries.^22^, ^23^ Demographic and outcome variables did not differ significantly between the NJ 2011–12 beneficiaries and matched non-NJ beneficiaries (data not shown).

### Pre-Storm

While some of the less severely impacted counties experienced a decrease in pre-storm hospital discharges, for the entire state there was a 7.1% relative increase in patients discharged during the week prior to Sandy’s landfall (from 51.8% to 55.5%, (P < 0.01)) as compared to the equivalent week in 2011. When extrapolated from the 20% subsample used for analysis, this increase corresponds to an estimated 295 additional Medicare FFS patients discharged. Almost half of the increase consisted of discharges to home health services (46%), followed by discharges to SNF (24%), and 17% discharged to home. A disproportionate number of discharges occurred in the more severely impacted counties, with a 10.7% relative increase of discharges in CHI Q_3_ and Q_4_ (from 50.4% to 55.8% of patients discharged, [P<.01]). This corresponds to an estimated extrapolation of 425 additional Medicare FFS patients discharged in these areas relative to the prior year. Hospital discharges in the week prior to Sandy’s landfall in CHI Q_4_ alone were not statistically different.

ALOS among discharged patients in the pre-storm week was 0.3 days shorter than that of DRG-matched NJ patients who were discharged at any time in 2011 or 2012 (P < 0.03). However, patients discharged the week prior to Sandy’s landfall did not have a statistically significant different CMI compared to the equivalent week in 2011, suggesting that factors other than clinical status may have influenced disposition decisions.

Compared to the equivalent week in 2011, hospital admissions through the ED resulting from a SNF-to-hospital transfer increased 38.9% (from 9.0% to 12.5%, [P<.01]) during the pre-storm period, corresponding to an additional 140 extrapolated admissions. Hospital admissions resulting from hospital-to-hospital or hospice-to-hospital transfers were not statistically different.

Patients transferred to hospitals from SNFs had DRG-specific CMI upon admission that were 32.7% higher in the week prior to landfall when compared to patients transferred from SNFs in the prior year’s equivalent week (1.40 vs. 1.96) (P<0.03). This suggests that more complex and/or more clinically ill patients were transferred from SNF-to-hospital during the pre-storm period. Of note, hospital admissions from inter-facility transfers did not vary significantly by CHI quartile (i.e., these occurred at a uniform rate statewide).

### Post-Storm

As shown in Table 2, there were 14.3% more ED visits among Medicare FFS beneficiaries in the state of NJ in the week following Superstorm Sandy when compared to previous week and equivalent weeks the year prior. Of these, 80% were discharged and 20% were admitted as inpatients. This observed split differed from the baseline where 60% of patients were discharged and 40% admitted. When extrapolated from the 20% subsample used for analysis, this represents an estimated increase of 1,558 ED visits for all Medicare FFS beneficiaries, of which 1,244 were discharged and 314 were admitted. The Monte Carlo simulation validation results provided in Table 2 show an even higher increase in ED utilization, suggesting that using NJ the year prior provides a conservative reflection of actual clinical practice. Finally, in contrast to the pre-storm week, there was not a significant increase in the proportion of ED admissions due to transfers from SNFs after the storm.

Geographically, the 14.3% surge in overall Sandy-related ED visits in NJ was not evenly distributed throughout the state. In fact, ED utilization actually decreased in the least impacted counties while it increased in the most impacted counties. In the hardest hit counties (those with CHI > 59), the surge was more than double (14.3% vs. 35.5%) the overall statewide surge, with these counties experiencing more than twice as many ED visits resulting in a discharge (19.1% vs. 44.7%) and three times as many ED visits resulting in an admission (7.2% vs. 21.4%) as compared to the state average. An estimated 1,187 additional Medicare FFS beneficiaries were seen in EDs in the Q_4_ counties with CHI > 59 in the week following Superstorm Sandy compared to the equivalent week of 2011 (Figure).

## DISCUSSION

This study represents a first step towards describing pre- and post-storm ED and hospital utilization behavior during 2012’s Superstorm Sandy in NJ. Our analysis of Medicare FFS claims suggests that NJ hospitals experienced multiple alterations in pre- and post-landfall patient movement for this population, including a significant surge in post-storm ED visits, associated with Sandy. In the week prior to the storm’s arrival, hospitals statewide discharged 7.1% more patients and coastal counties discharged almost 11% more patients than during the equivalent time period the prior year. The majority of these patients were discharged to home health services and SNFs. In addition, SNF-to-hospital transfers increased by almost 40%. While hospitals did not discharge more severely ill patients, our data show that patients transferred from SNFs and admitted to the hospital through the ED were significantly sicker than those transferred the equivalent week in 2011.

Although our data show that hospital discharges in NJ increased up to 11% in the week prior to Superstorm Sandy, the reasons for this utilization behavior are unknown. Hospitals may have been concerned about their ability to remain open, or concerned that the storm would impact staff ability to get to work. They may have anticipated a post-storm surge in patients. Or, patients may have been eager to get home before the storm and advocated for earlier release. Importantly, SNF-to-hospital transfers increased by 38.9%, and patients who were moved in the pre-storm period were significantly sicker than transfer patients in 2011 and the patients being discharged from the hospital. The data suggest that while hospitals were freeing up space, they were simultaneously managing a sicker inpatient population.

The top five hardest hit NJ counties experienced a 21.4% increase in inpatient admissions of patients covered by Medicare FFS. Identifying these at-risk individuals in a pre-disaster setting may improve their outcomes or even permit strategies to keep them out of the hospital altogether, similar to other studies that use Medicare data to identify electricity-dependent individuals (e.g., patients on dialysis) to improve disaster preparedness and response.^24^ Using existing claims data in this manner can contribute to promoting local, regional, and national health security without additional data-reporting requirements.

These findings have important implications for how individual facilities, healthcare systems, and healthcare coalitions consider planning for serious weather events with warning. During severe disasters, facilities should expect variable increases in patient surge depending on location, population demographics, infrastructural resiliency, and other factors and plan accordingly. Under the guidance of the U.S. Department of Health and Human Services Hospital Preparedness Program (HPP), healthcare systems are currently advised to maintain the ability to free up at least 20% of their routine bed census within four hours of a disaster.^25^ Our study provides empirical data in support of the need for facilities and healthcare systems to increase bed capacity in advance of severe weather events.

## LIMITATIONS

The study has several important limitations. First, the study uses a retrospective design, which is inherently subject to selection bias. Second, the analysis is limited to Medicare FFS beneficiaries and may not represent the experience of other populations including the privately insured, patients with Medicare Advantage, and patients with Medicaid. The continuous enrollment inclusion criterion selected for Medicare patients who did not die between 2011 and 2012, and as such may bias our findings to reflect a relatively healthier population. Finally, actual hospital occupancy at the time of the storm was not assessed (either by this research or at the time by NJ public health officials). According to an American Hospital Association report, NJ had an average occupancy rate of 70% in 2012, so additional capacity beyond that identified here as a result of hospital discharges may have existed.^26^ Daily hospital census data would be required to capture how many functional beds were available as the storm came ashore, but this cannot be extrapolated from Medicare data*.* Developing tools for measuring and communicating bed availability, occupancy, and associated surge capacity across healthcare coalitions in real time during such events will result in improved regional patient management.

## CONCLUSION

Meteorological projections increasingly suggest that the incidence and severity of extreme weather events are likely to increase by the end of the 21st century.^27^, ^28^ Sustainable and resilient healthcare facilities that are prepared to care for patients who may experience new or exacerbated health problems as a result of these extreme events is critical.^29^ In addition to weather-related events, other threats to the public’s health, including infectious diseases and injuries, require a health system prepared to respond. Unfortunately, there is little real-world, population-based evidence that describes the source, magnitude, and destination of the medical surge associated with natural or man-made disasters and public health emergencies. This evidence is important for promoting population health resilience, improving healthcare delivery during disasters, and identifying patients who may be at particularly high risk for adverse health outcomes. This study takes an important step in this direction, illustrating the relationship between the preparations for and medical impact of Superstorm Sandy in the state that bore the brunt of its landfall.

## Supplementary Information



## Figures and Tables

**Figure:**
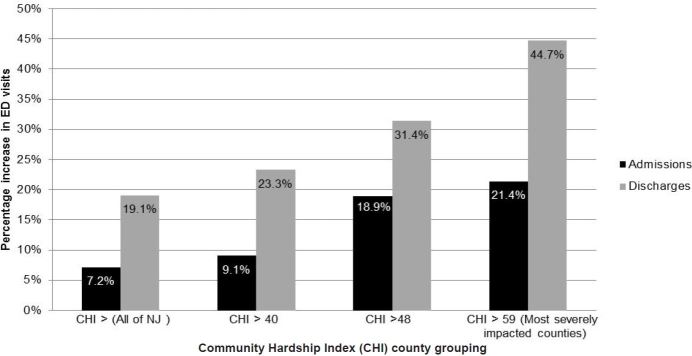
Percent increase in emergency department (ED) visits by Community Hardship Index (CHI) and ED disposition (2012 vs. 2011).

**Table 1:** Definition of county quartiles by Community Hardship Index (CHI).

Quartile (Q)	CHI	Number medicare FFS beneficiaries (N)	New Jersey counties
1^st^ (Q_1_)	< 39	37,581	Salem, Camden, Cumberland, Gloucester, and Burlington.
2^nd^ (Q_2_)	40–47	52,754	Warren, Passaic, Mercer, Cape May, Essex, and Atlantic.
3^rd^ (Q_3_)	48–58	56,193	Hudson, Bergen, Morris, Sussex, and Hunterdon.
4^th^ (Q_4_)	> 59	77,588	Union, Middlesex, Somerset, Ocean, and Monmouth.

*FFS,* fee-for-service.

**Table 2:** Estimated increase in emergency department (ED) utilization among New Jersey Medicare (fee-for-service) FFS beneficiaries by ED disposition in the week after Superstorm Sandy’s landfall.

Post-storm utilization	Actual increase in ED visits	Extrapolated increase in ED visits (all Medicare FFS beneficiaries)	Percent increase in ED visits (2011 vs. 2012)*	Percent increase in ED visits (Monte Carlo simulation)
ED visits	312	1,558	14.3%	17.7%
Discharged	249	1,244	19.1%	23.0%
Admitted	63	314	7.2%	8.2%

* Significant at p < 0.01. Percentage increase in ED visits discharges and admits do not sum up to the total percentage increase as each one is calculated separately.
