# Supplementary material for: Impact of Superstorm Sandy on Medicare Patients’ Utilization of Hospitals and Emergency Departments
Source: West J Emerg Med. 2017 Sep 21;18(6):1035–41. doi: 10.5811/westjem.2017.7.34730 (PMC5654871; doi:10.5811/westjem.2017.7.34730)
Supplement: Supplementary file 1 [file wjem-18-1035-s001.docx]

**APPENDIX.**

The Monte Carlo simulation (MCS) was used to model the probability of the increase in 2012 post-storm facility utilization outcome measures for the NJ Medicare FFS beneficiaries. This was accomplished by randomly matching 1:1 (with replacement) each NJ beneficiary demographic characteristics and ED utilization outcomes for the equivalent post-storm week in 2011 to 2011 beneficiaries from states not impacted by Sandy. Beneficiaries were matched on age, gender, and race; as well as ED utilization outcomes including no ED visit, ED visit resulting in an admission, and ED visit that was discharged. For each MCS iteration, randomly matched 2011 beneficiaries both in NJ and non-Sandy impacted states were tracked in 2012 and a difference-in-differences (DDD) regression was performed. The additional difference was between the number of visits one week before and week after landfall in 2012 as well as visits between the equivalent weeks in 2011 for the beneficiaries in NJ and non-Sandy affected states. MCS accounts for risk (unlike other techniques such as propensity score matching) by using different randomly matched control populations for each iteration and creating a probability distribution of the ED outcome variable of interest (Figure 2).

**Figure 2.** Monte Carlo simulation (MCS) generated probability distribution of increase in Emergency Department (ED) visits resulting in a discharge.
